# Visually assessed ischemia on cardiac magnetic resonance, but not quantitative perfusion metrics, predicts symptomatic improvement in coronary artery bypass

**DOI:** 10.1016/j.jocmr.2025.101953

**Published:** 2025-09-04

**Authors:** Tamim Akbari, Lukas Mach, Daniel J. Hammersley, Suzan Hatipoglu, Ruth Owen, Dylan Taylor, Joyce Wong, Shahzad G. Raja, Sunil K. Bhudia, Dudley J. Pennell, Brian P. Halliday, Richard E. Jones, Sanjay K. Prasad

**Affiliations:** a Royal Brompton and Harefield Hospitals, part of Guy’s and St Thomas’ NHS Foundation Trust, London, UK; b National Heart and Lung Institute, Imperial College London, London, UK; c King’s College Hospital NHS Foundation Trust, London, UK; d Royal Free London, NHS Foundation Trust, London, UK; e Department of Medical Statistics, London School of Hygiene and Tropical Medicine, London, UK; f Centro Nacional de Investigaciones Cardiovasculares, Madrid, Spain; g OXON Epidemiology, Madrid, Spain; h Anglia Ruskin University, Chelmsford, UK; i Essex Cardiothoracic Centre, Basildon, UK

**Keywords:** Cardiac magnetic resonance imaging, Coronary artery disease, Myocardial ischemia, Coronary artery bypass grafting

## Abstract

**Background:**

Serial perfusion cardiovascular magnetic resonance (CMR) in symptomatic patients undergoing coronary artery bypass grafting (CABG) may provide mechanistic insight into dynamic abnormalities of the myocardium.

**Objectives:**

To assess how changes in cardiac reperfusion and remodeling associate with symptom improvement in patients undergoing CABG

**Methods:**

Patients awaiting elective CABG completed serial quality of life questionnaires and detailed CMR at baseline and at 6–12 months post-CABG as per protocol. Automated fully quantitative stress and rest myocardial blood flow was calculated, alongside assessment of the visual ischemic burden. Findings were correlated with changes in symptomatology.

**Results:**

Of 40 patients who underwent serial evaluation with CMR (mean age 62.1 ± 9.3, median LVEF 68% [IQR: 62–73%]), there was improvement in the median visual ischemic burden (42% [IQR: 27–51] vs 18% [IQR: 11–21], P<0.001), mean global stress myocardial blood flow (1.34 ± 0.5 mL/min/g vs 1.59 ± 0.5 mL/min/g, P = 0.002) and median global myocardial perfusion reserve (1.85 ± 0.6 vs 2.4 ± 0.9, P<0.001) following CABG. Greater improvement in the SAQ-7 summary score was associated with a greater decrease in the visual ischemic burden following CABG (ρ = −0.38, P = 0.02). Quantitative MBF metrics did not associate with baseline or change in SAQ-7 summary score.

**Conclusion:**

Serial perfusion CMR identifies dynamic changes in markers of myocardial perfusion in patients following CABG. Greater reduction of visually assessed ischemia associated with improvement in SAQ-7 score. Quantitative perfusion indices were not associated with symptom improvement in this study. The results also suggest residual inducible ischemia post-CABG, requiring further studies to elucidate its clinical relevance.

## Graphical abstract

**Figure:**
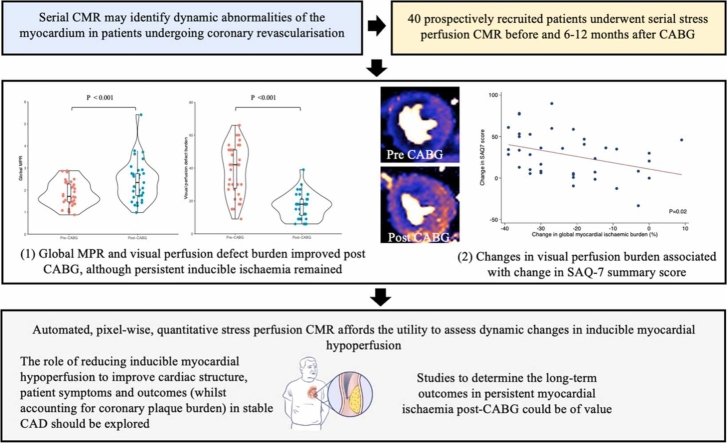
Myocardial hypoperfusion, assessed both qualitatively and quantitatively improved following CABG, with evidence of persistently impaired stress MBF. Visual perfusion defect burden, but not quantitative metrics associated with change in patient-reported health status. Despite residual ischaemia, most patients reported excellent health status following CABG. Studies to determine serial association between symptoms, quantitative CMR, and the long-term consequences of residual myocardial ischaemia should be considered. *CABG* coronary artery bypass grafting, *CMR* cardiovascular magnetic resonance, *MBF* myocardial blood flow, *MPR* myocardial perfusion reserve, *SAQ-7* Seattle Angina Questionairre-7.

## 1. Introduction

Coronary artery bypass grafting (CABG) offers a potential means of protecting against recurring myocardial ischemia and preventing the complications arising from the rupture of plaque in native vessels [1]. However, accurately predicting which patients will benefit from CABG presents a challenge; while offering longer term benefits, the risk of adverse outcomes following surgery in the short term remains high, and a significant number of patients continue to experience symptoms after the procedure [2], [3]. In the landmark ISCHEMIA trial, whilst there was no difference in major cardiovascular events, an initial invasive strategy (which included percutaneous coronary intervention [PCI] and CABG) led to greater symptom relief and a better quality of life for patients with angina at baseline as compared to the initial conservative strategy [4]. To determine key predictors of symptom improvement, comprehensive phenotyping using multiparametric cardiovascular magnetic resonance (CMR) holds promise by enabling the assessment of changes in contractile function, perfusion and myocardial tissue characteristics.

One key benefit of CMR is the ability to reproducibly investigate inducible hypoperfusion through automated assessment of myocardial blood flow (MBF) [5]. This approach extends beyond visually identifying perfusion defects, which may have utility when considering the correlation with symptoms [6] or evaluating multivessel disease. Traditional qualitative methods have shown reduced diagnostic accuracy in individuals with prior CABG [7] and currently, only single timepoint studies have evaluated the accuracy and prognostic value of fully quantitative techniques [8], [9]. Serial CMR datasets could enhance our understanding of the role of MBF calculation in assessing changes in myocardial perfusion and their correlation with symptom burden after CABG.

We conducted a prospective study to evaluate the correlation between serial changes in myocardial perfusion and anginal symptoms that accompany CABG in patients with CAD.

## 2. Methods

### 2.1. Study design

Between September 2019 and April 2021, an observational cohort study (The AMBITION study) was conducted, involving the prospective recruitment of patients with stable CAD awaiting CABG. Written informed consent was obtained from all patients. The study received approval from the National Research Ethics Service (19/EE/0166) and adhered to the principles of the Declaration of Helsinki. Blood samples were collected to assess levels of NT-proBNP and high-sensitivity Troponin I. The functional status of patients was evaluated using the New York Heart Association (NYHA) functional score, Canadian Cardiovascular Society (CCS) Angina grade, and Seattle Angina Questionnaire-7 at baseline and at follow-up. All patients underwent comprehensive CMR imaging, which included fully automated quantitative stress perfusion, parametric mapping, and late gadolinium enhancement imaging. In a subset of patients, transmural myocardial tissue biopsies were obtained from regions with and without inducible myocardial ischemia. The results of this sub-study have been published separately [10]. Serial evaluation, including functional assessment, blood tests, and CMR using an identical protocol, were performed at 6 months after CABG. Due to the effects of the COVID pandemic and a halt on research hospital visits, the study protocol was amended to allow an extension in follow-up of up to 12 months. Additionally, assessment of functional capacity using the 6-min walk test was stopped.

### 2.2. Inclusion and exclusion criteria

Patients awaiting clinically indicated CABG and evidence of inducible myocardial ischemia on CMR were eligible for inclusion. Exclusion criteria encompassed the following: a) valve disease necessitating intervention during surgery; b) evidence of concomitant non-ischemic cardiomyopathy; c) liver failure (e.g., international normalized ratio >2); d) contraindication to CMR; e) inability to consent.

### 2.3. CMR protocol

All CMR scans were performed using a 1.5 Tesla Siemens Aera scanner (Siemens Healthineers, Erlangen, Germany). The imaging protocol included localizers, balanced steady-state free precession (bSSFP) cine long axis imaging, native myocardial T1 mapping using a modified Look-Locker inversion recovery (MOLLI) 5(3)3 sequence at the basal and mid-ventricular level. Intravenous adenosine was infused in a stepwise protocol over 3–5 min at a rate of 140–210 mcg/kg/min to induce an adequate stress response. An adequate stress response was determined by assessing symptoms and haemodynamic response as per standard practice. During the first pass of a gadobutrol bolus (0.05 mmol/kg IV), short-axis slices were acquired at the basal, mid-ventricular, and apical levels for perfusion imaging. Inline perfusion software within the Gadgetron online reconstruction framework was used to generate automated perfusion maps, along with pixel-wise myocardial blood volume maps [5]. A top-up dose of 0.05 mmol/kg of IV gadobutrol was administered, followed by bSSFP short-axis cine imaging. Late gadolinium enhancement (LGE) imaging was performed using a free-breathing phase-sensitive inversion recovery (PSIR) motion-corrected (MOCO) sequence after a 10-min delay. Post-contrast T1 mapping was conducted after 20 min, followed by rest perfusion imaging. At 6–12 months after CABG, patients underwent the same CMR protocol, including the administration of the same adenosine dose and infusion duration.

### 2.4. CMR analysis

All CMR analysis was performed by an independent Level 3 CMR Operator (SH) using CVI42 software (Circle Cardiovascular Imaging, Calgary, Alberta, Canada). The analysis was conducted in batches, with pre- and post-operative cases presented in a random order. Ventricular volumes were indexed to body surface area. Native T1 values were calculated at the mid-ventricular septum, excluding regions with late gadolinium enhancement [11]. Myocardial perfusion was assessed both visually and quantitatively. Visual perfusion defect burden was determined on a segment-by-segment basis by identifying areas of reduced relative signal intensity. A semi-quantitative approach was used to quantify visual perfusion defect burden, following the methodology employed in the MR INFORM trial, which employed a 32-segment model [12], [13]. Quantitative analysis with transmural MBF was calculated for each segment on a pixel-wise basis. This approach used a blood tissue exchange model and partial differential equations, including estimation of the arterial time delay between the arrival of the contrast bolus in the left ventricle cavity and the pixel of interest [14]. Cases were reviewed by our blinded independent expert if required, and segments with infarct pattern LGE were excluded from the quantitative perfusion analysis. Global stress and rest MBF values were derived by averaging values across all non-infarcted segments at stress and rest, respectively, and myocardial perfusion reserve was subsequently calculated (MPR = Global stress MBF/Global rest MBF). Additionally, with emerging evidence for the potential prognostic role of rate pressure product (RPP) correction of myocardial flow reserve [15] and guideline recommendations for its consideration [16], we performed an analysis to calculate RPP-corrected MPR to account for the effects of resting haemodynamics. LGE quantification was performed using the Full-Width at Half Maximum methodology [17]. The equations for calculating left ventricular extracellular volume fraction (ECV), indexed extracellular matrix volume, and indexed cell volume and RPP corrected MPR are described in the Supplementary Figure 2.

### 2.5. Statistical analysis

#### 2.5.1. Power calculation

A patient sample size of 32 was calculated to yield a power of 84.1% to detect at least a moderate correlation of ρ≥0.50 at the 5% significance level [18], [19].

Continuous variables were reported as mean (standard deviation) or median (interquartile range [IQR]), while categorical variables were reported as N (%). Patient characteristics were compared between those with a global MPR below the median and those with a global MPR at or above the median using the 2-sample t-test for continuous variables and the chi-squared test or Fisher's exact test for categorical variables. Differences in symptoms, blood markers, and CMR metrics were summarized as mean or median (95% confidence interval) and compared between pre- and post-CABG using the paired t-test or Sign test, depending on the distribution of the changes in each variable. Spearman correlation coefficients were calculated to assess the associations between baseline or change in patient-reported health status and CMR metrics. All statistical tests used a two-sided significance level of P<0.05. The statistical analyses were performed using Stata v17.0.

## 3. Results

Of 45 patients, 43 individuals were enrolled and underwent serial examination with multiparametric CMR (mean age 62.1 ± 9.3, median LVEF 68% [IQR: 62–73%], median MPR 1.7 [1.4–2.2]). Overall, 42 patients (97.7%) had 2 or 3 vessel coronary disease, and 13 (30.2%) had a prior myocardial infarction. In 38 individuals (88.4%), 2 or more vessels were grafted. The majority of patients had one arterial graft (65.1%) (Table 1). There were eight cases of complete arterial revascularisation. Of the two patients that did not complete the study, one patient was lost to follow-up and one patient died. Supplementary Figure 1 details the derivation of the cohort.

**Table 1:** Baseline characteristics, stratified by global MPR

	All patients	Global MPR		P-value
		≤1.7	>1.7	
Demographics	N = 43	N = 19	N = 19	
Age (y)	62.1 (9.3)	62.6 (10.4)	61.5 (8.0)	0.69
Sex				0.69
Male	41 (95.3)	23 (95.8)	18 (94.7)	
Female	2 (4.7)	1 (4.2)	1 (5.3)	
Heart rate (bpm)	67.3 (11.8)	69.8 (11.3)	64.3 (11.9)	0.13
SBP (mmHg)	135.2 (20.4)	140.3 (20.6)	128.7 (18.8)	0.07
Number of severely diseased vessels				0.59
1	1 (2.3)	1 (4.2)	0 (0.0)	
2	14 (32.6)	6 (25.0)	8 (42.1)	
3	28 (65.1)	17 (70.8)	11 (57.9)	
Prior MI	13 (30.2)	7 (29.2)	6 (31.6)	0.86
Prior revascularisation	12 (27.9)	5 (20.8)	7 (36.8)	0.25
Hypertension	30 (69.8)	20 (83.3)	10 (52.6)	0.03
Diabetes mellitus	14 (32.6)	10 (41.7)	4 (21.1)	0.15
Hypercholesterolemia	34 (79.1)	19 (79.2)	15 (78.9)	0.99
Current smoker	3 (7.0)	1 (4.2)	2 (10.5)	0.41
Number of vessels grafted				0.08
1	5 (11.6)	1 (4.2)	4 (21.1)	
2	22 (51.2)	12 (50.0)	10 (52.6)	
3	16 (37.2)	11 (45.8)	5 (26.3)	
Number of arterial grafts used				0.52
0	1 (2.3)	1 (4.2)	0 (0.0)	
1	28 (65.1)	14 (58.3)	14 (73.7)	
2	13 (30.2)	8 (33.3)	5 (26.3)	
3	1 (2.3)	1 (4.2)	0 (0.0)	
Medications				
Betablocker	32 (74.4)	17 (70.8)	15 (78.9)	0.54
ACEi/ARB	29 (67.4)	17 (70.8)	12 (63.2)	0.59
Lipid-lowering medication	42 (97.7)	23 (95.8)	19 (100.0)	0.56
Calcium channel blocker	14 (32.6)	9 (37.5)	5 (26.3)	0.44
Long-acting nitrate	10 (23.3)	4 (16.7)	6 (31.6)	0.22

*ACEi* angiotensin-converting enzyme inhibitor, *ARB* angiotensin II receptor blocker, *DBP* diastolic blood pressure, *MI* myocardial infarction, *SBP* systolic blood pressure, *MPR* myocardial perfusion reserve. Continuous variables are reported as mean (standard deviation). Categorical variables are reported as N (%). Characteristics were compared between patients with a Global MPR <median versus those with a Global MPR ≥median using 2-sample t-tests for continuous variables and Fisher’s exact tests for categorical variables

The median difference between baseline and follow-up CMR was 238 days [IQR 201–387]. The median interval between baseline CMR to surgery was 14 days [IQR 6–85 days], and the median interval between CABG to follow-up CMR scan was 191 days [IQR 137–240 days].

At baseline, the median NYHA class was 2 [IQR: 1–2], CCS class was 2 [IQR: 1–3] and SAQ-7 Summary score (0–100, higher = less symptomatic) was 65.0 [IQR: 43.1–86.9]. The median indexed end-diastolic LV volume, indexed end-systolic LV volume and LVEF at enrollment were 70.6 mL/m^2^ [IQR: 65.3–80.3 mL/m^2^], 26.8 mL/m^2^ (± 15.4 mL/m^2^) and 68.4% [IQR: 62.2–73.0%], respectively. On quantitative perfusion imaging, the mean global stress MBF was 1.34 ± 0.49 mL/min/g, mean global rest MBF was 0.73 ± 0.15 mL/min/g, and mean global MPR was 1.85 ± 0.5. There was no difference in the dose of adenosine administered pre- and post-CABG [(mean dose 180.5 mcg/kg/min vs 183.6 mcg/kg/min; P = 0.94), (mean length of infusion 4 min 12 s vs 4 min 21 s; P = 0.71)] to provoke an adequate stress response. The median visual perfusion defect burden was 42.0% [IQR: 27.0–51.0%] prior to CABG. At baseline mean native septal T1 was 1005.0 [IQR 973–1044], ECV was 0.28 ± 0.04, indexed LV cell volume was 46.5 ± 8.9 mL/m^2^, and indexed LV extracellular matrix volume fraction was 18.2 ± 4.6 mL/m^2^. Patients with a lower global MPR were more likely to have history of hypertension (P = 0.03). All serial results are detailed in Table 2.

**Table 2:** Serial changes in symptoms, CMR metrics, and blood results following CABG

Variable	Pre-CABG	Post-CABG	Mean/Median* Difference	P-value
Symptom class				
NYHA functional score	2 (1, 2)	1 (1, 1)	−1 (−1, 0) *	<0.001
CCS angina grade	2 (1, 3)	1 (1, 1)	−1 (−1, −1) *	<0.001
SAQ−7 Summary score	65 (43, 86.9)	100 (89.2, 100)	30 (8.33, 36.4) *	<0.001
Anthropometric data				
HR (beats/min)	64.0 (11.0)	63.0 (10.0)	1.0 (−1.6, 3.6)	0.68
SBP (mmHg)	134.5 (21.6)	131.8 (22.8)	2.6 (4.4, 9.6)	0.62
BSA (m^2^)	2.06 (0.17)	2.07 (0.18)	0.01 (0.0, 0.024)	0.07
CMR characteristics				
LVEDVi (mL/m^2^)	70.6 (65.3, 80.3)	69.8 (60.5, 81.7)	−4.25 (−7.1, 0.9) *	0.07
LVESVi (mL/m^2^)	26.8 (15.4)	27.5 (14.5)	0.73 (−0.75, 2.22)	0.32
LVEF (%)	68.4 (62.2, 73)	65.2 (57.3, 72.9)	−2.23 (−5.73, 0.75) *	0.07
LAVi	34.4 (11.6)	39.6 (11.1)	5.28 (2.58, 7.98)	<0.001
LVMi	68.7 (12.9)	65.9 (12.5)	−2.75 (−4.7, −0.73)	0.009
3D GLS	−12.7 (−15.5, −11.0)	−13.0 (−16.0, −10.5)	−0.23 (−1.43, 0.25) *	0.13
3D GCS	−19.0 (−20.4, −15.5)	−17.9 (−19.9, −13.8)	0.47 (−0.21, 1.10) *	0.16
3D GRS	29.6 (23.3, 36.0)	28.8 (22.4, 36.3)	1.02 (−1.35, 2.89) *	0.36
Septal T1	1005 (973, 1044)	998 (959, 1032)	−11 (−25, 15) *	0.53
ECV fraction	0.28 (0.26, 0.29)	0.28 (0.26, 0.31)	0.002 (−0.008, 0.017) *	0.88
LV extracellular matrix volume	18.2 (15.6, 21.8)	18.0 (15.0, 20.2)	−0.35 (−1.98, 0.44) *	0.88
LV cell volume	46.5 (8.89)	44.4 (8.36)	−2.06 (−3.66, −0.459)	0.013
Global stress MBF (mL/min/g)	1.34 (0.49)	1.59 (0.50)	0.25 (0.099, 0.41)	0.002
Global rest MBF (mL/min/g)	0.73 (0.15)	0.69 (0.15)	−0.03 (−0.07, 0.01)	0.100
Global MPR	1.85 (0.55)	2.38 (0.87)	0.54 (0.28, 0.79)	<0.001
RPP Corrected Global MPR	1.55 (0.47)	1.85 (0.56)	0.30 (0.09, 0.50)	0.006
Global visual perfusion defect burden (%)	42 (27, 51)	18 (10.5, 21)	−22.5 (−30, −18) *	<0.001
Global stress endocardial/epicardial ratio	0.77 (0.08)	0.80 (0.08)	0.028 (−.009, 0.054)	0.05
Extent of MI scar (%)	7.6 (2.6–15.7)	7.0 (3.8–15.4)	1.0 (−1.5 to 2.0)	0.46
Blood results				
NTproBNP (ng/L)	256 (267)	311 (427)	54.9 (−33.7, 143)	0.22
hsTroponinl (ng/L)	13.4 (31.7)	8.74 (12.9)	−4.64 (−15.1, 5.79)	0.37

Continuous variables are reported as mean (standard deviation) or median (IQR). Categorical variables are reported as N (%). Serial characteristics were compared using the paired t-test for approximately normally distributed variables and the Sign test for non-normal data. The mean/median difference (95% CI) between the pre- and post-operative results is also detailed. The median differences are represented with *. *CABG* coronary artery bypass grafting, *CCS* Canadian Cardiovascular Society, *ECV* extracellular volume, *GCS* global circumferential strain, *GLS* global longitudinal strain, *GRS* global radial strain, *IQR* interquartile range, *LAD* left anterior descending artery, *LCx* left circumflex artery, *LAVi* indexed left atrial volume, *LVEDVi* indexed left ventricular end-diastolic volume, *LVEF* left ventricular ejection fraction, *LVESVi* indexed end-systolic LV volume, *LVMi* indexed left ventricular mass, *MBF* myocardial blood flow, *MPR* myocardial perfusion reserve, *NYHA* New York Heart Association, *RCA* right coronary artery, *RPP* rate pressure product, *SAQ-7* Seattle Angina Questionnaire-7

### 3.1. Serial changes to health status and circulating markers of myocardial wall stress and injury

There were improvements (baseline versus follow-up) in NYHA class (2 [IQR: 1–2] vs 1 [IQR: 1–1], P<0.001), CCS angina grade (2 [IQR: 1–3] vs 1 [IQR: 1–1], P<0.001) and SAQ-7 Summary score (65.0 [IQR: 43–86.9] vs 100.0 [IQR: 89.2–100.0], P<0.001) after CABG. Levels of NT-proBNP (256 ± 267 ng/L vs 311 ± 427, P = 0.22) and hsTroponin I (13.4 ± 31.7 ng/L vs 8.7 ± 12.9; P = 0.37) were similar post-operatively.

### 3.2. Serial changes to CMR assessment of myocardial perfusion

**Fig. 1:**
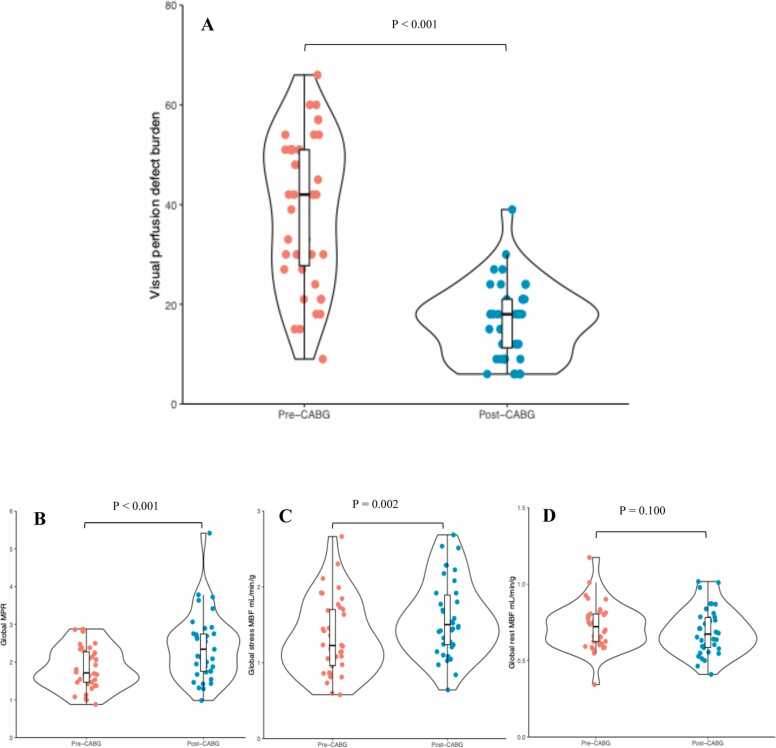
Serial CMR myocardial perfusion data in patients undergoing CABG. Plot demonstrating the serial reduction in visual perfusion burden (%) following CABG (1A). Plots demonstrating the increase in global MPR (1B) and stress MBF (1C) following CABG. There was no significant change in global rest MBF (1D). *CABG* coronary artery bypass grafting, *CMR* cardiovascular magnetic resonance, *MBF* myocardial blood flow, *MPR* myocardial perfusion reserve

Overall, 40 (93%) patients underwent serial perfusion imaging and 38 patients had serial MBF data for analysis. After CABG, the median burden of visual perfusion defect decreased (42.0% [27.0–51.0] at baseline vs 18.0% [10.5–21.0] at follow-up, P<0.001) (Fig. 1A). All patients, however, had evidence of stress perfusion defects following CABG, with 30 (75%) patients having a residual global perfusion burden of >10%. On quantitative perfusion imaging, there was an increase in mean global stress MBF (1.34 ± 0.49 mL/min/g at baseline vs 1.59 ± 0.50 mL/min/g at follow-up, P = 0.002) and median global MPR (1.71 [IQR:1.36–2.22] at baseline vs 2.40 [IQR: 1.72–2.75] at follow-up, P = 0.002) (mean global MPR baseline vs follow-up; 1.85 ± 0.5 vs 2.38 ± 0.90, P<0.001) following CABG (Figs. 1B and 1C). There was no change in mean global rest MBF post-CABG (0.71 ± 0.15 mL/min/g vs 0.69 ± 0.15 mL/min/g, P = 0.100, Fig. 1D). Mean global stress endocardial/epicardial ratio increased following CABG (0.77 ± 0.08 vs 0.80 ± 0.08, P = 0.05). No baseline characteristics were associated with a visual perfusion defect burden of >10% at follow-up (Supplementary Table 1).

### 3.3. Serial changes in tissue characterization, myocardial structure, and function

There was no change in mean native septal T1 (1005 ±  [IQR 973–1044] vs 998 [IQR 959–1032], P = 0.53), ECV fraction (0.28 [IQR 0.26–0.29] vs 0.28 [IQR 0.26–0.31], P = 0.88) or mean indexed LV extracellular matrix volume after CABG (baseline; 18.2 ± 4.6 mL/m^2^ vs follow-up; 18.0 ± 3.9 mL/m^2^, P = 0.88). There was a small decrease in indexed LV cell volume (46.5 ± 8.9 mL/m^2^ vs 44.4 ± 8.4 mL/m^2^, P = 0.01) after CABG.

There was no significant change in median indexed LV end-diastolic volume (70.6 mL/m^2^ [IQR: 65.3–80.3 mL/m^2^] vs 69.8 mL/m^2^ [IQR 60.5–81.7 mL/m^2^], P = 0.07), indexed LV-end-systolic volume (26.8 ± 15.4 mL/m^2^ vs 27.5 ± 14.5 mL/m^2^, P = 0.32) or LVEF (68.4% [IQR: 62.2–73.0%] vs 65.2% [IQR: 57.3–72.9%], P = 0.07) after CABG. Post-CABG indexed left atrial volume increased (baseline; 34.4 ± 11.6 mL/m^2^ vs follow-up; 39.6 ± 11.1 mL/m^2^, P<0.001).

### 3.4. Association between health status, myocardial perfusion, and tissue characterization metrics

**Fig. 2:**
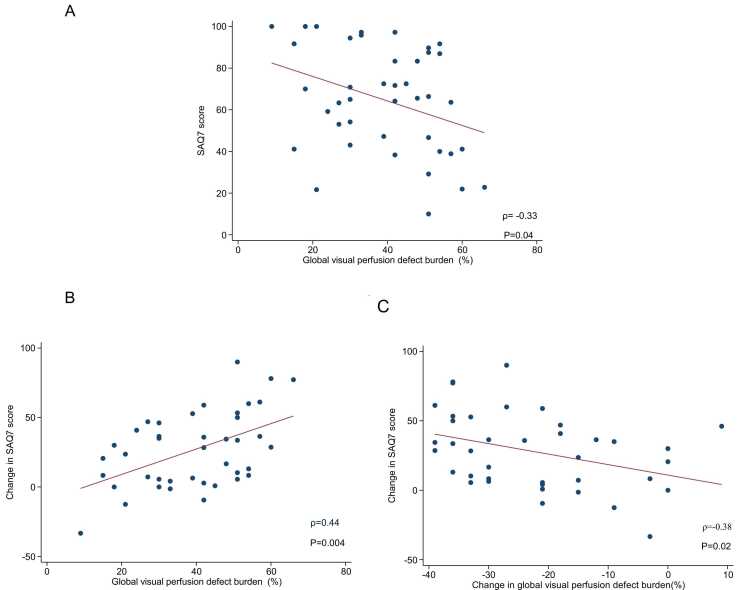
Associations between visual perfusion defect burden and SAQ-7 summary score. A: Plot demonstrating the association between greater baseline visual perfusion defect burden and lower baseline SAQ-7 summary score. B: Plot demonstrating the association between higher baseline global visual perfusion defect burden and greater change in SAQ-7 summary score. C: Plot demonstrating the association between change in global visual perfusion defect burden and change in SAQ-7 summary score. *SAQ* Seattle Angina Questionnaire

Increased baseline global visual perfusion defect burden was associated with a lower baseline SAQ-7 summary score (Range 0–100; higher =less symptomatic) (ρ = −0.33, P = 0.04, Fig. 2A). Additionally, a greater improvement in the SAQ-7 summary score was associated with a larger myocardial ischemic burden at baseline (ρ = 0.44, P = 0.004, Fig. 2B). Finally, a greater improvement in SAQ-7 summary score was associated with a larger decrease in global myocardial ischemic burden following CABG (ρ = −0.38, P = 0.02, Fig. 2C).

**Fig. 3:**
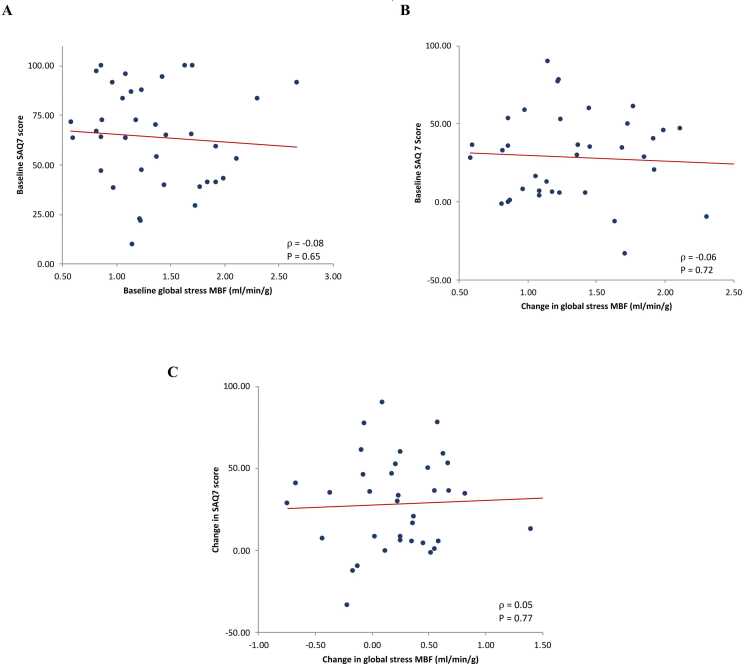
Associations between stress MBF and SAQ-7 summary score. A: Plot demonstrating the lack of association between SAQ-7 summary score and baseline stress MBF. B: Plot demonstrating the lack of association between change in SAQ-7 summary score and baseline stress MBF. C: Plot demonstrating the lack of association between change in SAQ-7 summary score and change in stress MBF. *MBF* myocardial blood flow, *SAQ* Seattle Angina Questionnaire

Neither baseline nor serial change in global stress MBF, global rest MBF, and global MPR were associated with baseline or change in SAQ-7 summary scores (Fig. 3, Supplemental Figs. 3 and 4). No association was present when MPR values were corrected for RPP (Supplemental Fig. 5). There was also no association between baseline global MPR and LVEF (ρ = 0.20, P = 0.22) or change in global MPR and LVEF (ρ = 0.22, P = 0.18). Similarly, no association was demonstrated with change in global MPR and change in 3D GLS on follow-up (ρ = 0.23, P = 0.15).

Baseline LGE extent (% of LV) did not correlate with baseline SAQ score (ρ = 0.19, P = 0.32) nor did change in LGE extent correlate with change in SAQ score on follow-up (ρ = 0.31, P = 0.11). Additionally, there was no significant association between change in LV extracellular matrix volume and baseline global myocardial ischemic burden (ρ = −0.24, P = 0.14). Finally, there was no significant association demonstrated between change in LV extracellular matrix volume and change in global myocardial ischemic burden (ρ = 0.25, P = 0.13, Supplementary Fig. 6B).

## 4. Discussion

In this study, we used serial multiparametric CMR to evaluate the association of dynamic changes in myocardial structure, function, and perfusion with patient-reported health status in patients undergoing CABG. The main findings are: i) Qualitative and fully quantitative measures of inducible myocardial ischemia by stress perfusion CMR improved after CABG; ii) Decrease in visual perfusion defect burden, but not stress MBF, associated with improvement in patient-reported health status following CABG; iii) Postoperatively, the majority of patients reported excellent health status despite 75% of patients having a residual visual ischemic burden of >10%.

### 4.1. Dynamic myocardial perfusion and tissue characterization indices following CABG

A number of nuclear studies have established the prognostic role for myocardial perfusion indices [16], [20], [21], [22] demonstrating improvement in these metrics following coronary revascularisation [23], [24], [25]. There remains a paucity of data, however, detailing the dynamic assessment of MBF in CABG cohorts; prior studies either reporting single timepoint assessment or excluding or recruiting only a limited number of post-graft individuals [26], [27], [28]. This current study details novel data describing the serial improvement in CMR-derived stress MBF and MPR in patients undergoing CABG, suggesting that quantitative stress perfusion CMR can detail myocardial blood flow during vasodilator stress in the presence of bypass grafts. This may have prognostic relevance, and a recent meta-analysis of 79 studies of patients undergoing invasive and non-invasive assessment of coronary flow, demonstrated that abnormal coronary flow was associated with all-cause mortality (HR 3.78, 95% CI: 2.39–3.99) and MACE (HR 3.42, 95% CI: 2.92–3.99). Only three studies included CMR-derived assessment of myocardial flow, with one study including patients with prior CABG [25]. Recent retrospective data, however, have demonstrated the independent association between CMR-derived MBF and outcomes in patients with prior CABG underlining the clinical relevance of perfusion metrics in these patients [9]. The lack of change in rest MBF observed in our study could be expected as a result of autoregulatory mechanisms responsible for maintaining myocardial blood flow constant at rest [29].

### 4.2. Association between patient-reported health status and myocardial perfusion

The association between improvement in the SAQ-7 summary score and decreasing visual perfusion defect burden post-CABG is intuitive and supports the strategy of restoring epicardial blood supply to reduce anginal symptoms. We observed a mean 26 point increase (95% CI 8.3–35.7; P<0.001) in SAQ-7 score post-CABG in keeping with previous studies, above the 5 point change that is considered clinically meaningful [30], [31]. The result also aligns with data from the recent ORBITA-2 trial which demonstrated the utility of percutaneous coronary intervention to improve health status in patients with coronary disease [32]. The data, however, highlights residual inducible myocardial ischemia in patients post-CABG, despite more than 75% of cases (34/44) achieving full anatomical revascularisation, building on previous studies using semi-quantitative perfusion CMR [33] and challenging the concept that CABG provides complete revascularization. However, despite residual inducible ischemia, the majority of patients reported excellent health status post-CABG (60% reporting summary SAQ-7 score of 100, 82% reporting a score of 75 or above). The lack of correlation between symptoms and residual inducible ischemia suggests there may be a limited role for routine CMR-derived ischemia assessment in asymptomatic patients post-CABG.

The lack of association between the quantitative perfusion metrics and SAQ-7 summary score is, however, interesting and could reflect an important degree of microvascular dysfunction in these patients; an entity which may associate less well with symptoms and is less easily appreciated on assessment of the raw first-pass perfusion imaging. Large validation studies of SAQ-7 in microvascular dysfunction are lacking. In one study of 259 women with suspected coronary microvascular dysfunction, coronary flow reserve did not associate with SAQ-7 score on multiple regression analysis [34]. In the recent ORBITA-COSMIC randomized controlled trial of coronary sinus reducer for angina relief, despite improvement in daily angina score, coronary sinus reducer did not improve stress MBF, suggesting a lack of correlation of symptoms with CMR-derived myocardial blood flow [35]. These results, however, are discordant with prior data, a recent observational study demonstrating the association between quantitative MBF on positron emission tomography and patient-reported angina [6].

Finally, multiple tissue characterization parameters of intracellular and extracellular composition were assessed by serial CMR analysis. Further studies are required to investigate these parameters in depth. Trials to identify modifiable parameters in patients with stable CAD that could represent novel drug targets would be of value.

## 5. Limitations

The study patients were enrolled in a single-center study, although this allowed for a standardized protocol. Graft patency was not assessed at follow-up and therefore the etiology of the persisting perfusion defects could not be determined (e.g., graft failure, progressive native vessel disease, or microvascular dysfunction). Future studies assessing the serial association between symptoms, quantitative CMR, and graft patency (e.g., using CT coronary angiography) would be of value.

Additionally, the use of adenosine stress perfusion in the context of bypass grafts has challenges both in technical execution as well as in the interpretation of results. Arterial input function (AIF), a critical parameter in perfusion imaging, is typically measured in the LV cavity. CABG provides multiple channels of myocardial perfusion (native vessels as well as arterial and venous grafts) with different flow characteristics (varying flow rates, vessel capacitance, and transit times) making it difficult to ascertain accurate AIF representative of the true input for all areas of the myocardium. We analyzed AIF peak gadolinium concentration (Full-Width at Half Maximum methodology) pre and post-CABG and found no significant difference at rest (peak concentration, mmolL^-1^, pre vs post-CABG; 5.97 ± 1.70 vs 6.07 ± 1.95; P = 0.84) and stress (5.27 ± 1.90 vs 5.58 ± 1.83; P = 0.31). The time to peak concentration was shorter post-CABG both at rest (Arterial input function first pass, seconds, pre vs post-CABG; 7.32 ± 2.60 vs 6.07 ± 1.38; P = 0.01) and stress (5.38 ± 1.99 vs 4.32 ± 1.17; P = 0.002). Despite these challenges prior studies evaluating the use of perfusion imaging in patients with prior CABG are encouraging [8], [9], [28], [36], [37]. The patients underwent unblinded CABG and therefore, the potential for the improvement in symptoms to be attributed, at least in part, to placebo effect is possible. Finally, strict blinding of the CMR analysis may not have been maintained due to the visibility of sternal wires despite removal of multi-slice HASTE and true FISP sequences. The CMR analysis was, however, performed by an independent reader performing the analysis, in batches with scans presented in a differing order.

## 6. Conclusion

In patients undergoing CABG, despite improvement in both qualitative and fully quantitative measures, visual assessment of perfusion defect burden on CMR, rather than derived MBF metrics, predicts change in symptoms, suggesting that the traditional assessment of CMR ischemia may have a more clinically relevant role compared to quantitative perfusion modules.

Additionally, we report excellent health status by patients despite post-operative inducible ischemia following CABG. Patients may show significant improvement in symptoms despite residual ischemia, suggesting a limited role for routine CMR-derived ischemia assessment in asymptomatic patients post-CABG. This should be factored in serial assessments of patients with multivessel coronary disease. Studies to determine the serial association between functional capacity, symptoms, quantitative CMR, and the long-term consequences of residual myocardial ischemia should be considered.

## Funding

This work was supported by a National Heart and Lung Institute Foundation grant awarded to Professor Sanjay Prasad and Dr Richard Jones. Dr Brian Halliday is supported by an Intermediate Clinical Research Fellowship from the 10.13039/501100000274British Heart Foundation (FS/ICRF/21/26019) and 10.13039/501100000833Rosetrees Trust. Dr Lukas Mach was supported by the British Society for Heart Failure Research Fellowship, British Heart Foundation Clinical Research Training Fellowship, Rosetrees Trust and Alexander Jansons Myocarditis UK.

## Author contributions

**Tamim Akbari:** Writing – review & editing, writing – original draft, resources, project administration, methodology, data curation, conceptualization. **Lukas Mach:** Writing – review & editing, writing – original draft, methodology, data curation. **Daniel J. Hammersley:** Writing – review & editing, methodology, investigation, data curation. **Suzan Hatipoglu:** Writing – review & editing, validation, methodology, formal analysis, data curation. **Ruth Owen:** Writing – review & editing, validation, software, methodology, formal analysis, data curation. **Dylan Taylor:** Data curation, formal analysis. **Joyce Wong:** Writing – review & editing, validation, software, resources, methodology. **Shahzad G. Raja:** Writing – review & editing, validation, data curation. **Sunil K. Bhudia:** Writing – review & editing, validation, supervision, data curation. **Dudley J. Pennell:** Writing – review & editing, validation, supervision, resources, formal analysis, conceptualization. **Brian P. Halliday:** Writing – review & editing, supervision, methodology, investigation, formal analysis, conceptualization. **Richard E. Jones:** Writing – review & editing, writing – original draft, project administration, methodology, formal analysis, data curation, conceptualization. **Sanjay K. Prasad:** Writing – review & editing, writing – original draft, validation, methodology, formal analysis, conceptualization.

## Declaration of competing interest

The authors declare the following financial interests/personal relationships which may be considered as potential competing interests: Lukas Mach reports financial support was provided by British Heart Foundation. Lukas Mach reports financial support was provided by Rosetrees. Richard Jones reports financial support was provided by Imperial College London National Heart and Lung Institute. Sanjay Prasad reports financial support was provided by Imperial College London National Heart and Lung Institute. Brian Halliday reports financial support was provided by British Heart Foundation. Dudley Pennell reports a relationship with Siemens Medical Solutions USA Inc that includes: consulting or advisory and funding grants. Brian Halliday reports a relationship with AstraZeneca Pharmaceuticals LP as an advisor. The other authors declare that they have no known competing financial interests or personal relationships that could have appeared to influence the work reported in this paper.
