# Supplementary material for: Visually assessed ischemia on cardiac magnetic resonance, but not quantitative perfusion metrics, predicts symptomatic improvement in coronary artery bypass
Source: J Cardiovasc Magn Reson. 2025 Sep 4;27(2):101953. doi: 10.1016/j.jocmr.2025.101953 (PMC12730852; doi:10.1016/j.jocmr.2025.101953)
Supplement: Supplementary file 1 — Supplementary material [file mmc1.docx]

# Supplementary materials

# Supplementary Tables

| **Supplemental Table 1. Predictors of significant inducible ischaemia at follow-up** | | |
| --- | --- | --- |
| **Variable** | **Univariable** | |
|  | **OR (95% CI)** | **P-value** |
| **Age (per 10 years)** | 0.98 (0.45, 2.13) | 0.96 |
| **BMI (kg/m2)** | 0.86 (0.71, 1.04) | 0.13 |
| **Diabetes mellitus** | 2.00 (0.36, 11.23) | 0.43 |
| **Hypertension** | 0.43 (0.08, 2.41) | 0.34 |
| **Current smoker** | -- |  |
| **NYHA** |  | 0.90 |
| 1 | Reference group |  |
| 2 | 1.41 (0.25, 7.90) |  |
| 3 | 0.87 (0.13, 5.82) |  |
| **CCS** |  | 0.73 |
| 1 | Reference group |  |
| 2 | 1.33 (0.22, 8.22) |  |
| 3 | 0.75 (0.13, 4.36) |  |
| **SAQ-1 summary score** | 1.00 (0.97, 1.03) | 0.86 |
| **LVEF (%)** | 1.07 (0.98, 1.16) | 0.11 |
| **LGE mass** | 0.93 (0.85, 1.02) | 0.11 |
| **Number of arterial grafts used** |  | 0.87 |
| 0 | Reference group |  |
| 1 | 1.29 (0.26, 6.37) |  |
| 2 | -- |  |
| 3 | -- |  |
| Univariable associations between baseline characteristics and residual inducible ischaemia, defined as global visual perfusion defect burden>10% at follow-up.  BMI = body mass index; CCS = Canadian Cardiovascular Society; LGE = late gadolinium enhancement; LVEF = left ventricular ejection fraction; NYHA = New York Heart Association; SAQ-7 = Seattle Angina Questionnaire-7. | | |

| **Supplementary Table 2. Univariable associations between baseline characteristics and change in global MPR.** | | |
| --- | --- | --- |
|  | **Univariable** | |
| **Variable** | **Coefficient (95% CI)** | **P** |
| **Age (per 10 years)** | 0.02 (-0.25, 0.28) | 0.91 |
| **BMI (kg/m2)** | 0.02 (-0.05, 0.08) | 0.60 |
| **Diabetes mellitus** | 0.02 (-0.52, 0.56) | 0.94 |
| **Hypertension** | 0.15 (-0.36, 0.66) | 0.56 |
| **Current smoker** | -0.24 (-1.33, 0.84) | 0.65 |
| **NYHA** |  | 0.77 |
| 1 | Reference group |  |
| 2 | -0.09 (-0.67, 0.50) |  |
| 3 | 0.10 (-0.56, 0.77) |  |
| **CCS** |  | 0.73 |
| 1 | Reference group |  |
| 2 | 0.14 (-0.46, 0.74) |  |
| 3 | -0.10 (-0.73, 0.53) |  |
| **SAQ-1 summary score** | 0.01 (-0.00, 0.02) | 0.25 |
| **LVEF (%)** | 0.01 (-0.02, 0.04) | 0.69 |
| **LGE mass** | -0.03 (-0.06, 0.00) | 0.08 |
| **Number of arterial grafts used** |  | 0.65 |
| 0 | Reference group |  |
| 1 | 0.31 (-1.24, 1.85) |  |
| 2 | 0.54 (-1.05, 2.13) |  |
| 3 | -0.21 (-2.36, 1.94) |  |

| **Supplementary Table 3.** Univariable associations between baseline characteristics and change in global stress MBF. | | |
| --- | --- | --- |
|  | **Univariable** | |
| **Variable** | **Coefficient (95% CI)** | **P** |
| **Age (per 10 years)** | 0.11 (-0.05, 0.26) | 0.18 |
| **BMI (kg/m2)** | -0.01 (-0.05, 0.03) | 0.65 |
| **Diabetes mellitus** | 0.11 (-0.21, 0.44) | 0.48 |
| **Hypertension** | 0.18 (-0.12, 0.49) | 0.23 |
| **Current smoker** | -0.30 (-0.95, 0.35) | 0.36 |
| **NYHA** |  | 0.46 |
| 1 | Reference group |  |
| 2 | 0.06 (-0.30, 0.41) |  |
| 3 | 0.15 (-0.25, 0.55) |  |
| **CCS** |  | 0.72 |
| 1 | Reference group |  |
| 2 | 0.11 (-0.26, 0.47) |  |
| 3 | 0.07 (-0.31, 0.45) |  |
| **SAQ-1 summary score** | 0.00 (-0.00, 0.01) | 0.53 |
| **LVEF (%)** | 0.01 (-0.01, 0.03) | 0.20 |
| **LGE mass** | -0.03 (-0.04, -0.01) | <0.001 |
| **Number of arterial grafts used** |  | 0.91 |
| 0 | Reference group |  |
| 1 | 0.18 (-0.77, 1.13) |  |
| 2 | 0.18 (-0.80, 1.16) |  |
| 3 | -0.04 (-1.36, 1.28) |  |

# Supplementary Figures

| 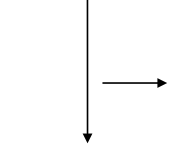  3 patients did not undergo follow up perfusion scanning due to patient preference or clinical reasons  2 patients had an alternative perfusion sequence  40 patients underwent serial perfusion-CMR  38 patients had serial MBF data for analysis |
| --- |
| Supplementary Figure 1: AMBITION study cohort  Derivation of the study cohort. CABG = coronary artery bypass grafting; CMR = cardiovascular magnetic resonance; PCI = percutaneous coronary intervention. |

| 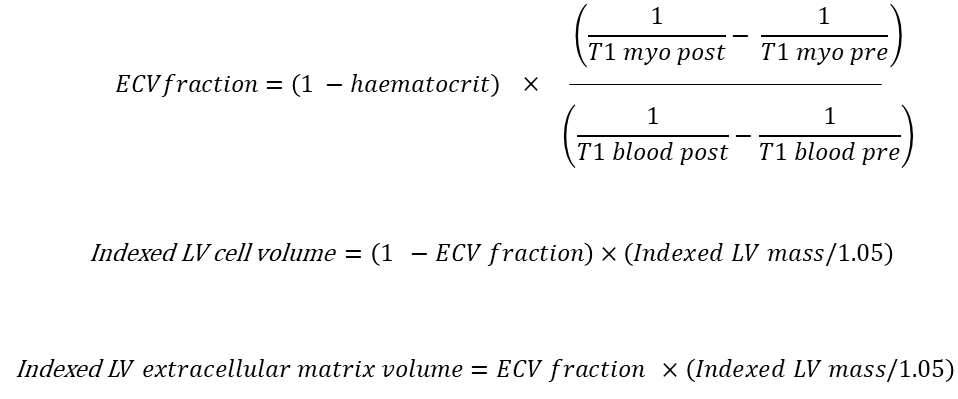  *RPP corrected Rest MBF = (Rest MBF / HR x SBP) x10,000*  *RPP corrected MPR = Stress MBF / RPP corrected Rest MBF* |
| --- |
| **Supplementary Figure 2: Equations**  Equations to calculate ECV, Indexed LV cell volume, Indexed LV extracellular matrix volume and RPP corrected MPR  ECV = extracellular volume; HR = heart rate; LV = left ventricular; MBF = myocardial blood flow; MPR = myocardial perfusion reserved; RPP = rate pressure product; SBP = systolic blood pressure |

**B**

**A**

**Supplemental Figure 3. Association of global rest MBF and SAQ-7 summary scores**

Plots show no statistically significant association between baseline SAQ7 score and baseline global rest MBF (plot A), change in SAQ7 score on follow up and baseline global rest MBF (plot B) or change in SAQ 7 score and change in global rest MBF (plot C)

MBF = myocardial blood flow; SAQ = Seattle angina questionnaire

**C**

**B**

**D**

**C**

**A**

**Supplemental Figure 4. Association of global MPR and SAQ-7 summary scores**

Plots show no statistically significant association between change in SAQ7 score and change in global MPR (plot A), change in SAQ7 score and baseline global MPR (plot B), baseline SAQ7 score and baseline global MPR (plot C) and change in SAQ7 score and change in global MPR when only transmural infarcted segments were excluded (plot D)

MPR = myocardial perfusion reserve; SAQ = Seattle angina questionnaire

**Supplemental Figure 5. Association of RPP corrected global MPR and SAQ-7 summary scores**

Plots show no statistically significant association between baseline SAQ7 score and RPP corrected global MPR (plot A), change in SAQ7 score and baseline RPP corrected global MPR (plot B), change in SAQ7 score and change in RPP corrected global MPR (plot C) and change in SAQ7 score and change in RPP corrected global MPR when only transmural infarcted segments were excluded (plot D)

MPR = myocardial perfusion reserve; RPP = rate pressure product; SAQ = Seattle angina questionnaire

**D**

**C**

**B**

**A**

| **A**  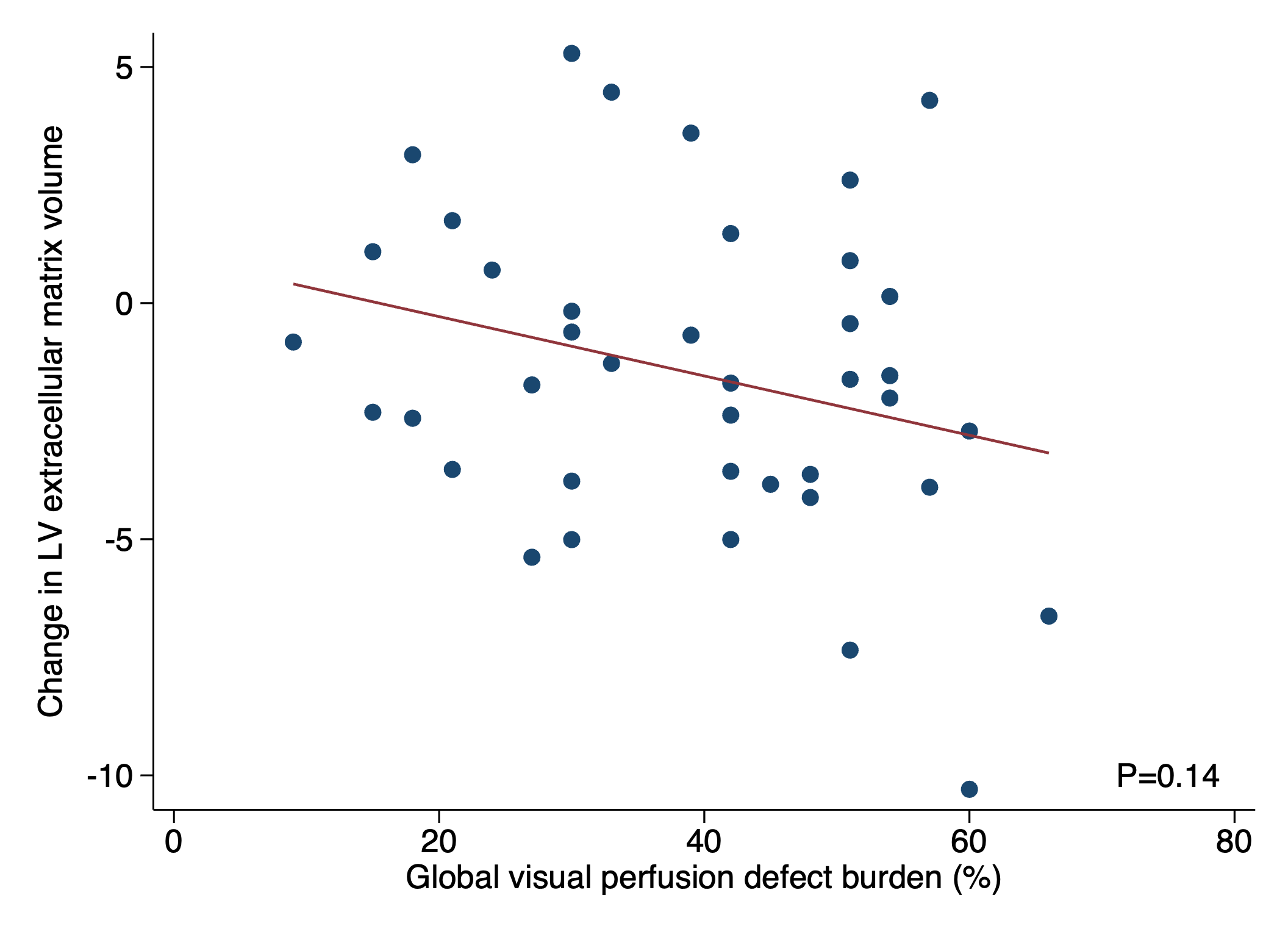 | **B**  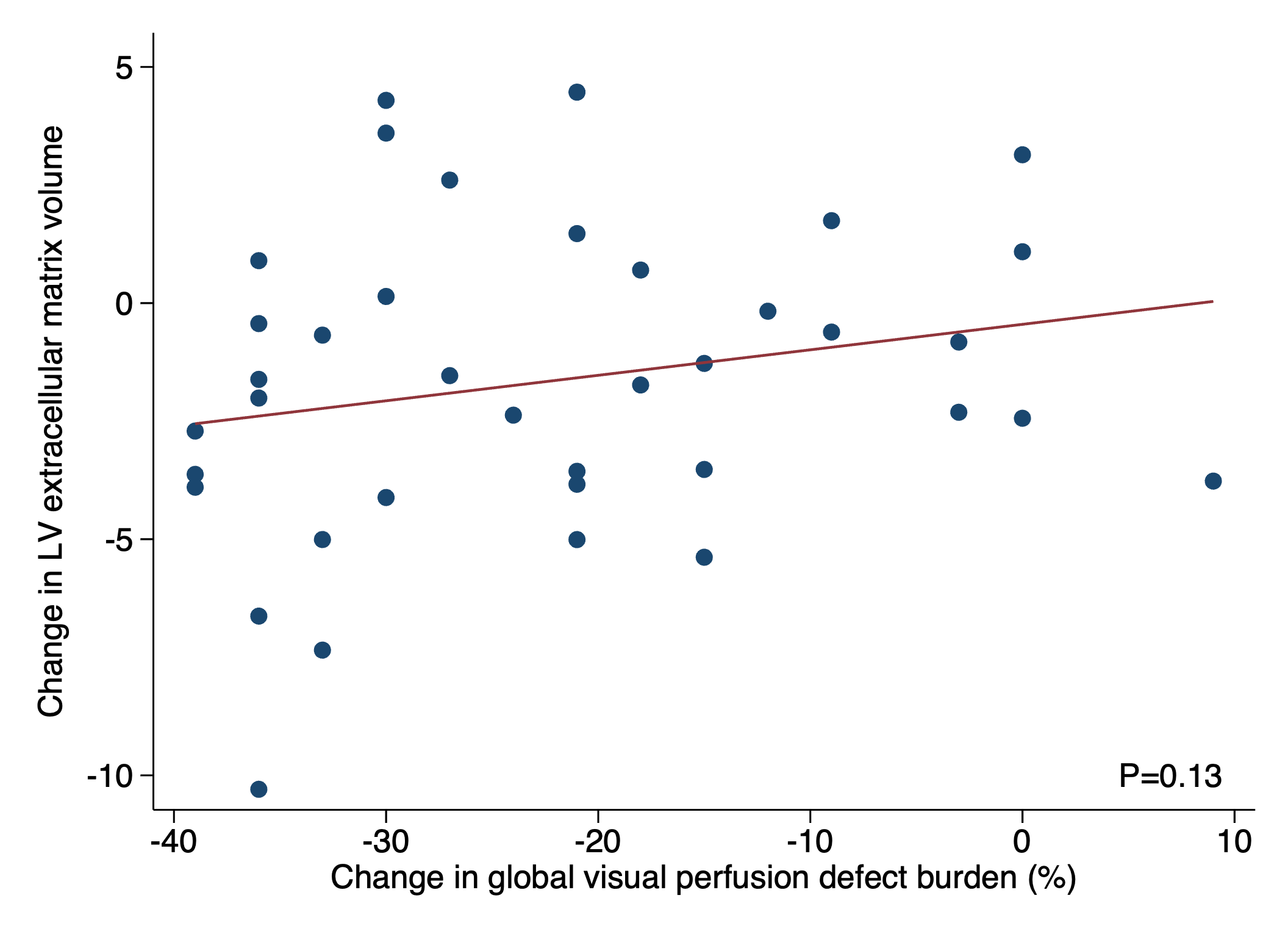 |
| --- | --- |
| Supplementary Figure 6: Association between visual perfusion defect burden and LV extracellular matrix volume.  A: Plot demonstrating the lack of significant association between change in LV extracellular matrix volume and baseline visual perfusion defect burden. B: Plot demonstrating the lack of significant association between change in LV extracellular matrix volume and change in visual perfusion defect burden.  LV = left ventricular. | |
